# Rapid enlargement of a traumatic pulmonary pseudocyst following an intraoperative leak test: a case report

**DOI:** 10.1186/s44215-023-00064-z

**Published:** 2023-07-03

**Authors:** Hiroki Matsumiya, Kanji Tanaka, Yuki Tahara, Masataka Mori, Masatoshi Kanayama, Akihiro Taira, Shinji Shinohara, Masaru Takenaka, Koji Kuroda, Fumihiro Tanaka

**Affiliations:** The Second Department of Surgery, School of Medicine, University of Occupational and Environmental Health, Yahatanishi, Kitakyushu, 807 Japan

**Keywords:** Traumatic pulmonary pseudocyst, Traffic trauma, Positive pressure ventilation, Check valve, Bronchopulmonary venous fistula

## Abstract

**Background:**

Traumatic pulmonary pseudocysts are a rare complication resulting from pulmonary contusion associated with blunt chest trauma.

**Case presentation:**

A 17-year-old male patient underwent emergency surgery for hemostasis of a traumatic lung injury caused by a car accident. Because the bleeding was from the peripheral lung, partial resection was performed with an automated suture machine. A routine leak test (positive pressure ventilation at 20 cm H_2_O) was performed as a precautionary measure. Postoperative imaging revealed rapid enlargement of a comorbid traumatic pulmonary pseudocyst. The collapse of the obliterating bronchus due to the accident and the intraoperative positive pressure ventilation for the air leak test were considered to have caused the rapid enlargement of the traumatic pulmonary pseudocyst. A thoracoscopic right lower lobectomy was performed on postoperative day 5. The patient had an uneventful postoperative course.

**Conclusions:**

To our knowledge, this is the first report of a rapidly enlarging traumatic pulmonary pseudocyst captured on preoperative and postoperative computed tomography. It would serve as a learning point for respiratory surgeons. These results suggest that when patients with traumatic pulmonary pseudocysts are operated on under general anesthesia, excessive positive pressure ventilation may cause a rapid expansion of the pulmonary pseudocyst.

**Supplementary Information:**

The online version contains supplementary material available at 10.1186/s44215-023-00064-z.

## Background

Traumatic pulmonary pseudocysts are a rare complication of blunt trauma. Greening et al. first reported this complication in 1957 [1], and Santos and Mahendra [2] proposed the term “traumatic pulmonary pseudocyst.” Several mechanisms have been hypothesized for the development of traumatic pulmonary pseudocysts after blunt trauma. Most authors agree that shearing forces cause lung lacerations, which enable air or fluid to escape into the tissue to produce a pseudocyst [3]. However, the exact mechanism underlying the exacerbation of traumatic pulmonary pseudocysts following their initial development has not been elucidated. Herein, we report a case of a traumatic pulmonary pseudocyst and its management.

## Case presentation

A healthy 17-year-old male patient with no significant medical history was brought to our hospital for emergency treatment of a contusion of the right chest sustained in a motorcycle accident. Contrast-enhanced computed tomography (CT) revealed a fracture in the right fifth rib, collapsed right lung, hematogenous pleural effusion, pulmonary contusion in the middle and lower lobes of the right lung, traumatic pulmonary pseudocyst, and liver injury (which was eventually followed up).

**Fig. 1:**
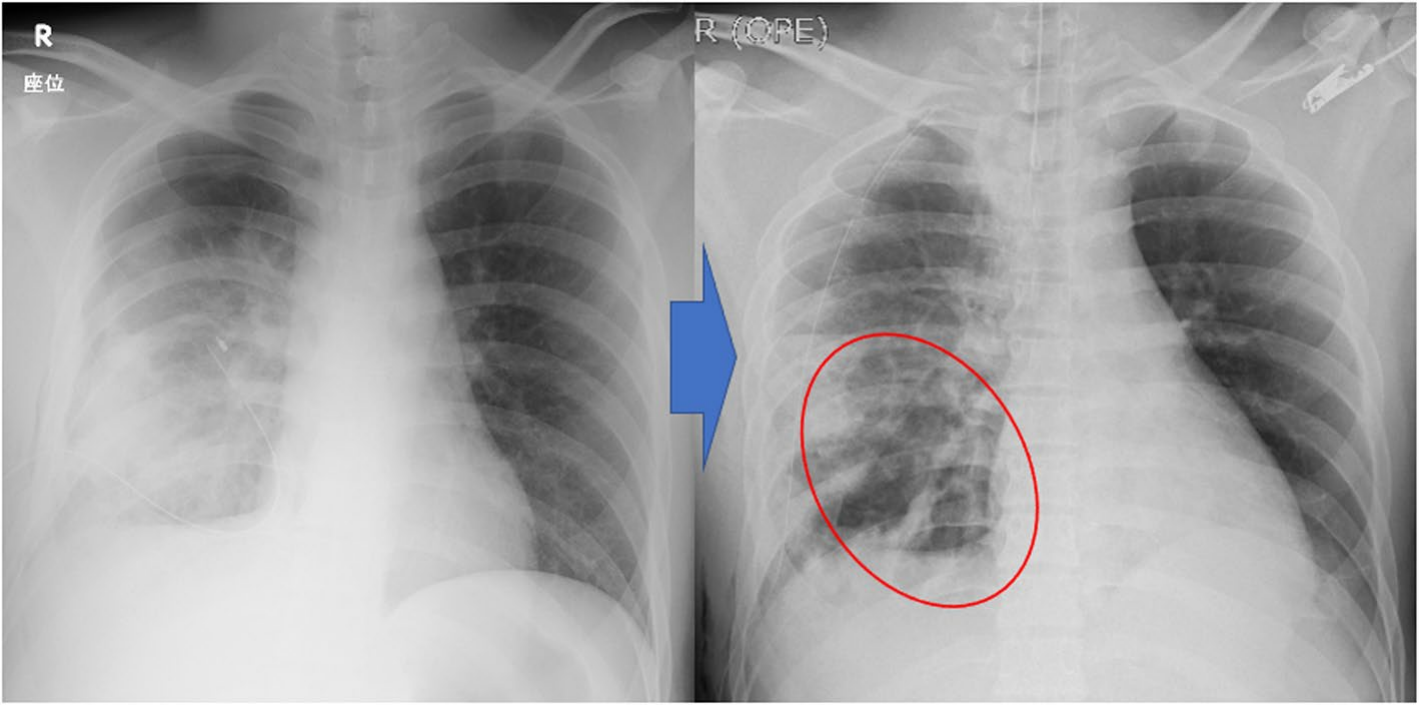
Pre- and postoperative changes in the radiographs. The postoperative radiograph shows a prominent cavity shadow in the right lower lung field (red oval)

**Fig. 2:**
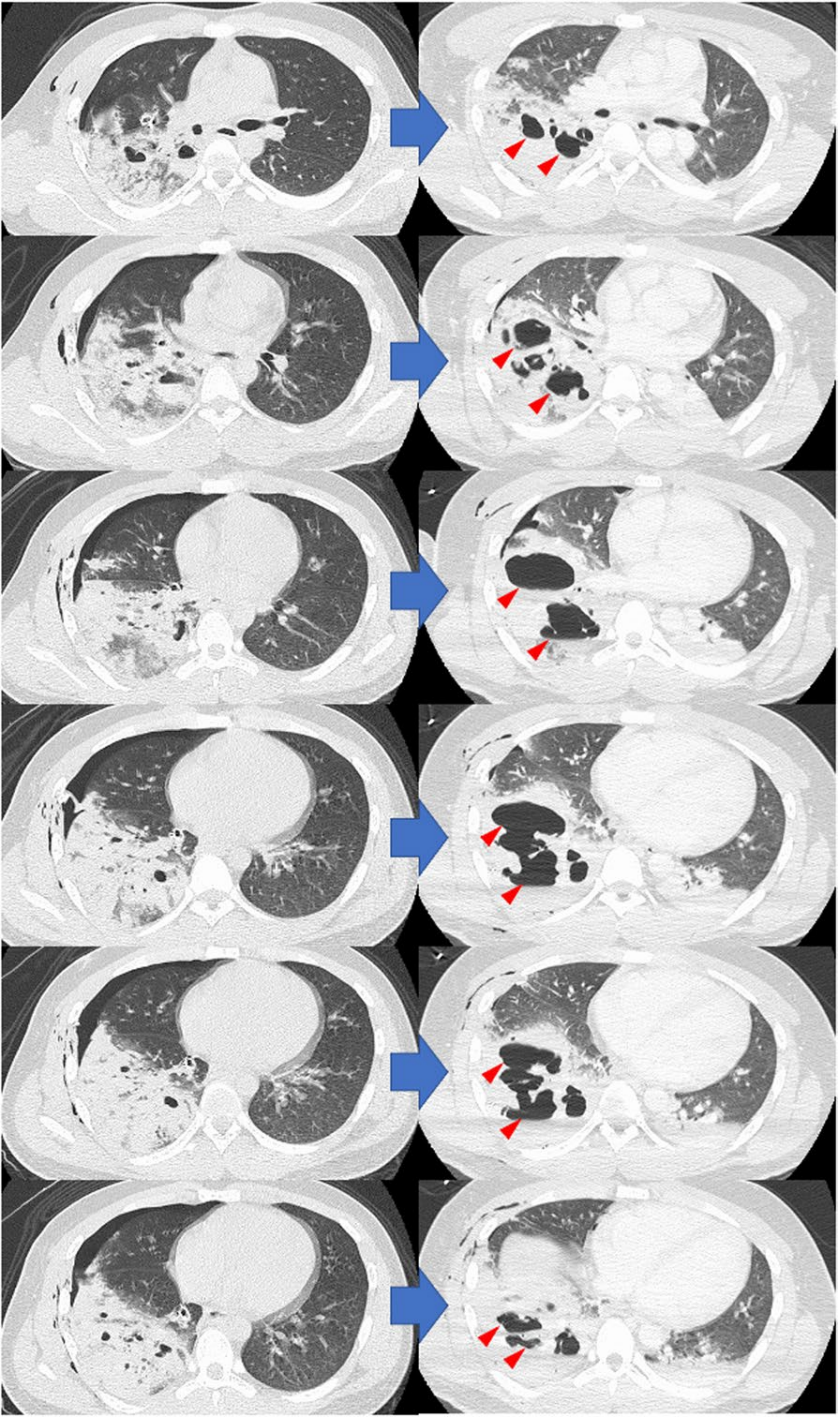
Computed tomography images before and after surgery. The traumatic pulmonary pseudocyst is markedly enlarged (red arrow). The cyst had a maximum diameter of 11 cm and was tense. The mediastinum was also slightly deviated to the left

We instituted continuous hematogenous drainage (Fig. 1 left, Fig. 2 left) and decided to perform emergency surgery to stop the bleeding and examine the thoracic cavity. On intraoperative visualization, we noted pleural defects in the middle and lower lobes from where the bleeding occurred. Since the bleeding was in the obliterated lung, a partial lung resection was performed using an automated suture machine. A leak test (20 cm H_2_O) was performed intraoperatively (Additional file 1).

Although hemorrhage and air leak were under control, a radiograph obtained immediately after the surgery detected a markedly enlarged cavity in the contused lower lobe of the right lung (Fig. 1 right). Bronchoscopy revealed no obvious bronchial damage. CT imaging was performed with careful use of an Ambu bag while the patient was still intubated. CT revealed a markedly enlarged cavity (Fig. 2 right).

The collapse of the obliterating bronchus caused by the accident and the intraoperative positive pressure ventilation for the air leak test were believed to have caused the rapid increase in the lung cyst size. The patient was admitted to the intensive care unit with intubation management (continuous positive airway pressure, positive end-expiratory pressure: 5 cm H_2_0, pressure support: 10 cm H_2_0) and weaned off the ventilator the next day. Although there was no significant increase in the cyst size afterward, the cyst was so large that we were concerned that it might develop into a tension pulmonary cyst.

**Fig. 3:**
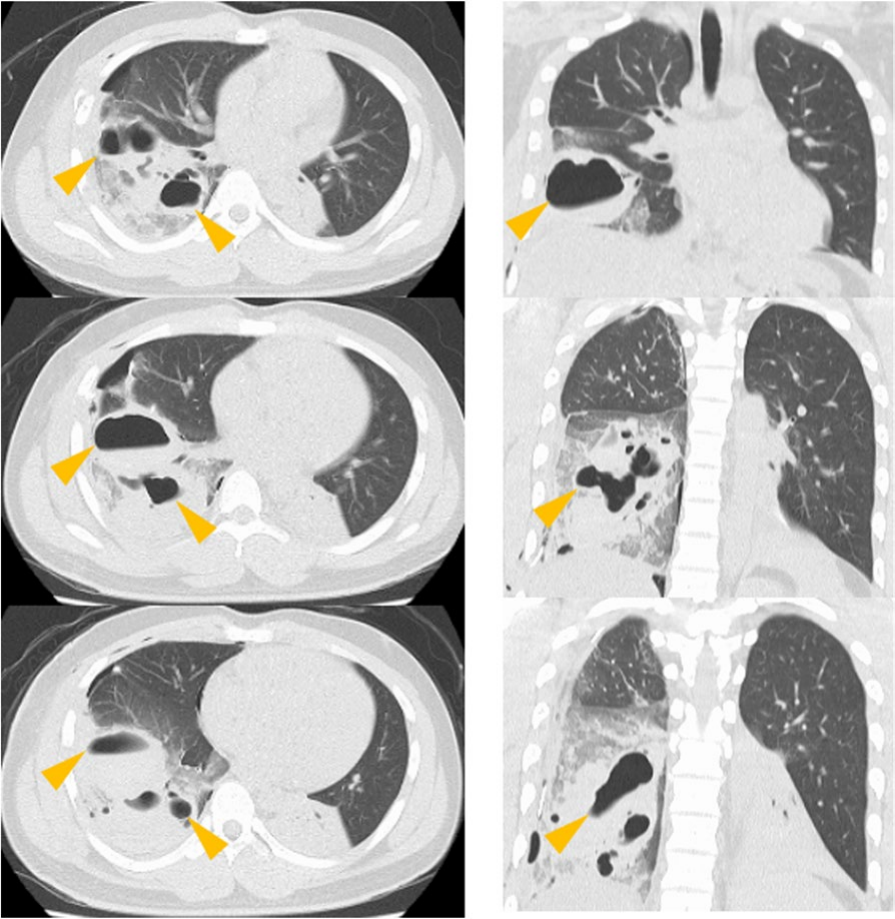
The CT image was taken 5 days after the first surgery (before reoperation). Left: horizontal section. Right: coronal section. Traumatic pulmonary pseudocyst remained unchanged and was tense (yellow arrow)

According to Melloni et al.’s treatment algorithm [4], managing it conservatively was possible. However, the cyst was large, with a maximum diameter of 11 cm, and CT images showed cyst tensions and a slight leftward migration of the mediastinum. As Kesieme et al. [5] reported, there was a risk of cerebral embolus. Therefore, after consulting with the pediatrician, and with the understanding of the patient and his family, we performed a reoperation (thoracoscopic right lower lobectomy) on postoperative day 5 (Fig. 3).

The patient’s postoperative course was good; the thoracic drain was removed on the 7th day after the initial surgery, and the patient was discharged home on the 12th day after the initial surgery. Two years have passed since the surgery, and the patient’s activities of daily living are unaffected.

## Discussion and conclusions

In this case, we believe the initial surgery had to be performed to control bleeding from the traumatic lung injury. However, we observed that the pulmonary pseudocyst was markedly enlarged immediately after the initial surgery. To our knowledge, this is the first report of a patient with a traumatic pulmonary pseudocyst with significant changes in the pulmonary pseudocyst on preoperative and postoperative CT images.

Intraoperative bronchoscopy showed no obvious bronchial injury, and pathological findings after lower lobectomy indicated no obvious check valve. The clinical course suggested that there was an injury to the obliterating bronchus. Retrospectively, we believe that the leak test (positive pressure ventilation at 20 cm H_2_O) at the time of the initial surgery was responsible for the marked enlargement of the pulmonary pseudocyst.

When performing general anesthesia during surgery, in the presence of a traumatic pulmonary pseudocyst, positive pressure ventilation should not be used unnecessarily. In cases with trauma caused by road accidents, non-anatomical changes may occur, and in surgeries with traumatic pulmonary pseudocysts, exacerbations such as in this case may occur. Therefore, respiratory surgeons need to be aware of this condition. In this case, the leak test should not have been performed “just in case.”

In addition, if the patient develops a tension pulmonary pseudocyst, there is a risk of air embolization associated with bronchopulmonary venous fistula. Thus, the procedure for administering hypertonic oxygen and low head position should be determined intraoperatively in collaboration with the anesthesiologist [5].

Melloni et al. [4] wrote, “Our experience confirms some articles in the literature that report the favorable outcome of TPPs without specific treatment. An accurate diagnosis is imperative to avoid unnecessary procedures, such as percutaneous drainage of an uncomplicated lesion and, in particular, unwarranted surgery.” In this case, the treatment algorithm used by Melloni et al. [4] could have been captured conservatively. However, the cyst was large, with a maximum diameter of 11 cm, and CT images showed tension of the cyst and a slight leftward shift of the mediastinum. There was a risk of cerebral embolism, as reported by Kesieme et al. [5]. Therefore, after consultation with the pediatrician and with the understanding of the patient and his family, we performed a reoperation (thoracoscopic right lower lobectomy) on postoperative day 5. If the leak test had not been performed at the time of the initial surgery, the pulmonary pseudocyst enlargement might have been more conservative.

In conclusion, this report describes a rare case. We could compare preoperative and postoperative CT images of a traumatic lung injury with a traumatic pulmonary pseudocyst rapidly increasing in size caused by routine positive pressure ventilation with 20 cm H_2_O for leak testing during surgery for bleeding control. These results suggest that when patients with traumatic pulmonary pseudocysts undergo surgery under general anesthesia, excessive positive pressure ventilation may cause rapid expansion of the pulmonary pseudocyst. If traumatic pulmonary pseudocysts become tense, their rapid enlargement also carries the risk of air embolization associated with bronchopulmonary venous fistulas. Hence, an anesthesiologist must be consulted in such cases.

## Supplementary Information

**Additional file 1: Video 1.** Intraoperative findings. Pleural defects and bleeding in the middle and lower lobes. As the bleeding was in the obliterated lung, partial lung resection was performed using an automated suture device. A leak test (20 cm H_2_O) was performed. No obvious leaks were noted. The patient was ventilated again with one lung; however, lung collapse was somewhat scant.

## Data Availability

The datasets used and/or analyzed during the current study are available from the corresponding author upon reasonable request.

## Abbreviation

CT: Computed tomography
